# Identification of *Quorum Sensing* Molecules of N-Acyl-Homoserine Lactone in *Leptospira* Strains Supernatants

**DOI:** 10.3390/microorganisms14081806

**Published:** 2026-08-16

**Authors:** Luz Olivia Castillo-Sánchez, Alejandro de la Peña-Moctezuma, Gerardo Uriel Bautista-Trujillo, Everardo Tapia-Mendoza, Adriana Romo-Pérez, Sergio Martínez-González, Fidel Avila-Ramos, Carlos Alfredo Carmona-Gasca

**Affiliations:** 1 *Leptospira* and Leptospirosis Research Group, Teaching, Research and Extension Center for Animal Production in the Plateau, School of Veterinary Medicine and Zootechnics, National Autonomous University of Mexico, Tequisquiapan 76795, Queretaro, Mexico; olicastillos@yahoo.com.mx; 2 Chiapas Medicinal Plants Research Institute, Meritorious Autonomous University of Chiapas, Tuxtla Gutiérrez 29050, Chiapas, Mexico; gerardo.trujillo@unach.mx; 3 Institute of Chemistry, National Autonomous University of Mexico, Coyocan, Mexico City 04510, Mexico; etapia@iquimica.unam.mx (E.T.-M.); adriana.romo@iquimica.unam.mx (A.R.-P.); 4 Laboratory of Functional Biology, Academic Unit for Veterinary Medicine and Zootechnics, Autonomous University of Nayarit, Compostela 63702, Nayarit, Mexico; sergio.martinez@uan.edu.mx; 5 Veterinary and Zootechnics Department, Division of Life Sciences, University of Guanajuato, Irapuato 36500, Guanajuato, Mexico; ledifar@ugto.mx

**Keywords:** AHL, autoinducers, Spirochetes

## Abstract

The bacterial *Quorum Sensing* system refers to the recognition of signaling molecules called autoinducers produced by bacteria when a certain cell density is reached in the environment. Those cell-density-dependent autoinducers regulate and coordinate diverse functional processes, such as bioluminescence, biofilm production, sporulation, and even the expression of some virulence factors, among others. There is a wide variety of autoinducers, and for Gram-negative bacteria, the canonical autoinducers are the N-acyl-homoserine lactones (AI-1). Presently, the production of autoinducers in *Leptospira* has not been described; therefore, the objective of this study was to detect and identify autoinducers in this bacterial genus. We report here the expression of AI-1 in cultures ≥2.4 × 10^8^ of *Leptospira meyeri*. Ethyl acetate extracts of *Leptospira* culture supernatants were capable of activating the β-galactosidase system in the biosensor *Agrobacterium tumefaciens* strain NTL4. Partial identification of the leptospiral supernatant extracts was done by thin-layer chromatography (TLC), showing a similar retention factor to the synthetic standard N-Octanoyl-DL-homoserine lactone (C8-AHL) in the *Leptospira* supernatant extracts. In addition, infrared spectroscopy (IR) analysis showed peaks corresponding to the lactone and amide groups in both the C8-AHL standard and the *Leptospira meyeri* culture extracts. Moreover, High-Performance Liquid Chromatography–Mass Spectrometry (HPLC-MS/MS) confirmed the same retention time (10.7 ± 0.1 min) in both the *Leptospira meyeri* supernatant extracts and the C8-AHL standard. These results show that *Leptospira meyeri* synthesizes N-acyl homoserine lactone family autoinducers, particularly the N-Octanoyl-DL-homoserine lactone, and lay the groundwork for future research on *Quorum Sensing* systems in *Leptospira*.

## 1. Introduction

*Leptospira* is a genetically diverse genus of Spirochetes, now reclassified into 68 species grouped into four subclades, instead of the groups historically termed saprophytic (S1 and S2), intermediate (P2), and pathogenic (P1) [1,2]. In parallel, serological classification in more than 300 serovars is also used, and a few phenotypic tests, such as virulence in animal models, growth rate at 30 °C, 37 °C or 14 °C, and growth in the presence of the purine analog 8-azaguanine, have been used to separate pathogenic from saprophytic strains [3,4]. Despite recent advances in understanding *Leptospira* and leptospirosis, which may be comparable to knowledge of other, better-known diseases [5], several aspects of the disease and the biology of the microorganism remain poorly understood or scarce.

Several *Leptospira* virulence factors have been described, including endoflagella that allow pathogenic *Leptospira* to cross cellular barriers and haematogenic spread; adhesins to bind to various host cell substrates, including the extracellular matrix; lipoproteins LipL32, LipL41, LipL36, and Loa22 that could potentially interact with host cells and the immune system during haematogenic spread. Lipopolysaccharide (LPS) that escapes recognition by TLR4 is recognized by TLR2 instead in human cells; hemolysins, leucine-rich repeat protein domains and peptidases, known to be associated with virulence; glycolipoproteins, peptidoglycan, heat shock proteins and flagellin [6]. Finally, biofilm formation in environmental settings favors *Leptospira* survival and dissemination, as well as infection in the animal hosts [7]. However, the mechanisms regulating the expression of these virulence factors have not yet been fully described.

Bacterial survival in the environment depends on the interaction with environmental conditions as well as a diverse number of organisms. They must adapt to such environmental conditions, and to do so, bacteria have developed their own communication mechanisms, such as the so-called *Quorum Sensing* system (QS). QS is an intercellular communication mechanism that regulates gene expression based on the recognition of signaling molecules produced by bacteria, depending on cell density (*Quorum*) [8,9]. QS has been described in both Gram-positive and Gram-negative bacteria, including humans, animals, and plant pathogens, along with non-pathogenic bacteria. It has been determined that QS is involved in the regulation of the expression of numerous biological processes, including production of virulence factors and exoenzymes, induction of the sporulation process, antibiotic resistance, bioluminescence, conjugative DNA transfer, control of plasmid copy numbers, biofilm formation, and the exopolysaccharide production [10,11,12].

The prototype of this system in Gram-negative bacteria is the LuxI/LuxR QS of *Aliivibrio fischeri* (formerly known as *Vibrio fischeri*) [13]. Such a system is composed of a synthase (protein LuxI), an autoinducer N-acyl homoserine lactone (AHL) QS signal, which at a time is sensed by the transcriptional regulator (LuxR) [14,15]. The AHL is produced at a basal level by bacteria, and its concentration increases when bacterial growth reaches the exponential phase. When the autoinducer reaches the threshold concentration, it enters the cell again and binds stably to the transcriptional regulator LuxR, activating the process of gene transcription and expression for a specific function regulated by the QS [16,17].

AHL-type autoinducers are derived from carbohydrate and fatty acid metabolism. Structurally, they are composed of a homoserine lactone ring linked to a fatty acid of the hydrocarbon chain (fatty acyl chain) by an amide bond. The hydrocarbon chain varies in length between 4 and 18 carbon atoms; it can be saturated or unsaturated, and may have oxo (O) or hydroxy (OH) group substitutions or no substitutions (hydrogen H) at the third carbon of the acyl chain. These characteristics provide variation and specificity to the QS in a heterologous bacterial population [16,18].

Currently, no studies on *Quorum Sensing* in *Leptospira* have been carried out so far; therefore, the objective of this study was to determine the production and identification of AHL autoinducer in *Leptospira*.

## 2. Materials and Methods

Microorganisms, preservation, and culture conditions

*Agrobacterium tumefaciens* NTL4 was used as a biosensor. The strain was preserved at −20 °C in Luria–Bertani broth (BD Difco^®^, Franklin Lakes, NJ, USA) containing gentamicin 30 µg/mL (LBg) and 30% glycerol (Sigma-Aldrich Merck^®^, Darmstadt, Germany). Propagation of *A. tumefaciens* was performed on LBg agar plates. For the detection assays, a colony was transferred to 5 mL LBg broth and incubated at 30 °C in agitation at 300 rpm for 12 h up to an OD_600_ of 1.8 to 1.9.

Three different *Leptospira meyeri* strains were used: the reference strain Veldratem 173 serovar Semaranga (kindly donated by the Oswaldo Cruz Institute (Fiocruz), Rio de Janeiro, Brazil), and two isolates, Tepetiltic and EcoPark, obtained from water bodies in Nayarit State, Mexico (this work). The three strains were preserved in semisolid Fletcher medium by subculture every three months and cultured at 30 °C in EMJH-Plus and Stuart (BD Difco^®^) liquid media (enriched with 10% rabbit serum) until they reached a concentration of 2.4 × 10^8^ leptospires/mL or higher. All biological experiments were conducted in triplicate.

Autoinducer extraction and detection

Before extraction, *Leptospira* cultures were observed under darkfield microscopy, and a purity test was performed to discard any bacterial contamination on blood agar plates incubated at 37 °C for 72 h.

The autoinducer molecules were extracted from the supernatant of the culture using a previously described method [19], with some modifications. Briefly, the extracts were prepared from 5 mL cultures; leptospires were removed by centrifugation at 10,000 rpm for 20 min; the supernatant was transferred to a new tube and extracted three times with equal volumes of 0.1% acetic acid (Sigma-Aldrich Merck^®^) acidified ethyl acetate (Sigma-Aldrich Merck^®^). The mixture was vortexed for 2 min, and the two phases were allowed to separate. The three volumes of ethyl acetate (organic phase) were pooled and evaporated to dryness at 45 °C; the obtained residues were dissolved in 100 µL of a 70/30 (*v*/*v*) methanol: water (Sigma-Aldrich Merck^®^) mixture and stored at −20 °C until use.

The autoinducer detection bioassays were performed in Petri dishes. Briefly, 20 mL of the *Agrobacterium tumefaciens* NTL4 biosensor strain culture was used to inoculate 80 mL of warm LBg medium containing 0.9% soft agar, 30 µg/mL gentamicin, and 20 mg of 5-bromo-4-chloro-3-indolyl-β-D-galactoside (X-gal) (Sigma-Aldrich Merck^®^) (20 mg/mL stock solution in dimethylformamide Sigma-Aldrich Merck^®^). The 20 mL agar culture solution was immediately poured into Petri dishes. After solidification, samples and controls were placed onto sterile 0.5 mm-diameter filter paper disks. As positive controls, 800 nM N-Octanoyl-DL-homoserine lactone (C8-AHL) (Sigma-Aldrich Merck^®^) and 7 µM N-Decanoyl-DL-homoserine lactone (C10-AHL) (Sigma-Aldrich Merck^®^) were used in 7 µL volumes. As a negative control, 7 µL of saline solution (0.9% NaCl) was used, and 7 µL samples of each *Leptospira* extract were used. Petri dishes were incubated at 30 °C for 12–24 h, protected from light in a closed plastic container.

The standard curve for semi-quantification of unsubstituted C8-AHL by the *A. tumefaciens* NTL4 biosensor strain was obtained by the agar diffusion method with concentrations ranging from 1 nM up to 500 µM, and the synthetic C8-AHL autoinducer dissolved in methanol (J. T. Baker^®^, Radnor, PA, USA). Diameters of the X-gal hydrolysis were measured and recorded; the values represented the means of three replicates and were analyzed using Pearson’s correlation coefficient [20].

Thin-layer chromatography (TLC) analysis

10 µL of *Leptospira* supernatant extracts and the unsubstituted synthetic standards N-Hexanoyl-DL-homoserine lactone C6-AHL (100 µM) (Sigma^®^), C8-AHL (10 µM) (Sigma^®^), and C10-AHL (200 µM) (Sigma^®^) were applied on the 20 cm × 20 cm aluminum-backed RP C18 silica sheets (reverse phase) (TLC Silica gel 60, RP-C18 F_254_s) (Merck Millipore^®^, Darmstadt, Germany). A methanol:water (80:20, *v*/*v*) mobile phase was used for the chromatography run. The TLC plate was dried at room temperature for 2 h in a fume hood until the solvent was evaporated.

For detection, the dried plates were overlaid with an *Agrobacterium tumefaciens* NTL4 strain culture as follows. 8 mL of the biosensor strain culture was used to inoculate 32 mL of warm LBg medium containing 0.9% soft agar, 30 µg/mL gentamicin, and 400 µL X-gal (20 mg/mL stock solution in dimethylformamide SigmaSigma-Aldrich Merck^®^). The culture mix was thoroughly homogenized, and a uniform overlay was immediately poured onto the surface of the TLC plate, allowed to solidify at room temperature protected from light in a closed plastic container, and then incubated at 30 °C for 12–24 h. Following incubation, the plate was examined for the presence of blue spots, indicative of AHL production, using the GelAnalyzer 26.1.1 software (https://gelanalyzer.com/). The synthetic standards were used as markers to allow estimation of putative AHL by comparing their retention factor (Rf = Distance traveled by the compound X/Distance traveled by the eluent Y) to that of the standards.

Partial purification of autoinducer molecules

Partial purification of the putative autoinducer molecules was performed by TLC for a given sample; 3 μL of the concentrated *Leptospira* culture extract was applied to a TLC plate in parallel with the C8-AHL standard. The remainder of the concentrated sample was applied to a second TLC plate as a series of four to six spots along the baseline; the two plates were run simultaneously in the same chamber.

The plate with the extract and the C8-AHL standard was covered with a culture of *A. tumefaciens* NTL4 as described above. Using the known retention factor (Rf), the area corresponding to the compound of interest was identified on the second plate, and the C18 silica plate matrix was scraped off, combined, and collected into a 2 mL microtube. The matrix was extracted three times with 1 or 2 mL of ethyl acetate acidified with 0.1% acetic acid; the three volumes of ethyl acetate were pooled and clarified by centrifugation at 10,000 rpm/5 min. The organic phase was left to evaporate to dryness at 45 °C, and the residue was redissolved in 100 µL of methanol (HPLC-grade, J. T. Baker). The extracts were stored at −20 °C until use. Finally, the biological activity of the autoinducer molecules recovered from the TLC plates was verified by an agar diffusion bioassay using the *A. tumefaciens* strain NTL4 biosensor.

Infrared Spectroscopy (IR)

Infrared spectra were recorded on a Nicolet iS50 FTIR spectrometer from Thermo Scientific (Massachusetts USA) by the attenuated total reflectance (ATR) technique [21,22]. Analyses were carried out in the mid-infrared region (4000–400 cm^−1^), and the spectra were collected at 36 scans with a resolution of 4 cm^−1^. All sample spectra were methanol-subtracted.

High-Performance Liquid Chromatography–Mass Spectrometry (HPLC-MS/MS).

The samples were analyzed by an HPLC-ESI-MS/MS Agilent apparatus model 1260-6530 QToF (Agilent Technologies, California USA ). The column used was a Zorbax Extend C-18 3.0 mm × 50 mm, 2.7 µm, and the temperature was set to 30 °C with a constant flow rate of 0.4 mL/min. Solvent A consisted of water with 0.1% formic acid, and solvent B was 100% acetonitrile. Chromatography was conducted using the following gradient elution conditions: t = 0 min 1% B; t = 3 min 11% B; t = 18 min 25% B; t = 25 min 100% B. The mass spectrometer was used in the Auto MS/MS mode. The spectrometer was operated in the positive mode, with electrospray ionization, a gas temperature of 275 °C, and a flow rate of 7 L/min, with the instrument held at a constant pressure of 40 psi and voltage of 4500 V, and a collision voltage of 30 eV.

## 3. Results

### 3.1. Autoinducer Detection

**Figure 1:**
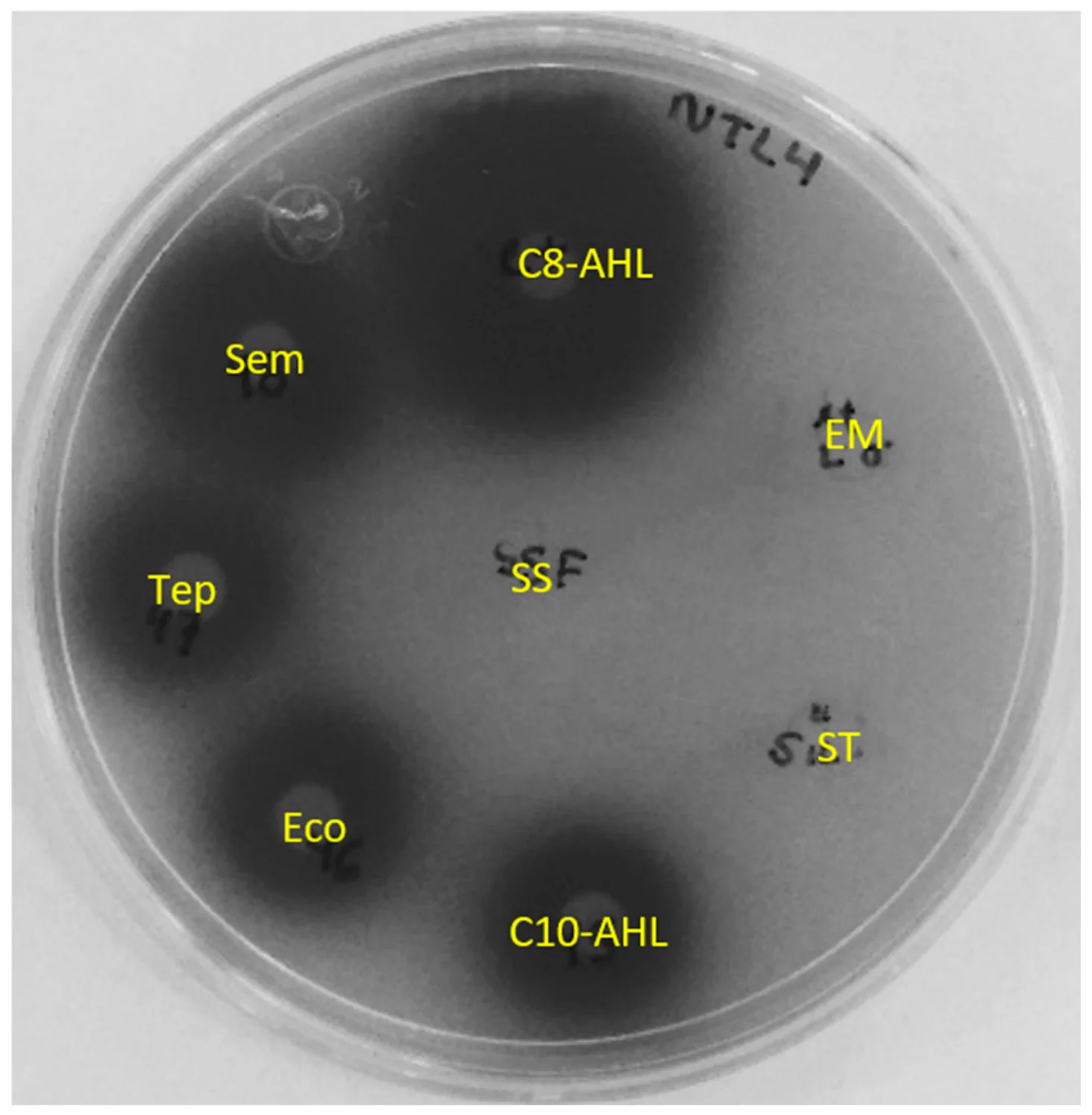
Detection of AHLs in the supernatant extracts of *Leptospira* cultures, using the *A. tumefaciens* strain NTL4 as biosensor on soft LBg agar plates (0.9%). SS: Saline solutions (negative control, 7 µL). C8-AHL 800 nM (30 mm Ø). C10-AHL 7 µM (18 mm Ø). Eco: *L. meyeri* strain EcoPark 7 µL (16 mm Ø). Tep: *L. meyeri* strain Tepetiltic 7 µL (18 mm Ø). Sem: *L. meyeri* strain Semaranga 7 µL (20 mm Ø). EM: EMJH-Plus uninoculated culture medium. ST: Stuart uninoculated culture medium. Ø: Diameter of the AHL diffusion ring in millimeters detected by X-gal hydrolysis.

*A. tumefaciens* strain NTL4 was capable of detecting autoinducer molecules in culture extracts of the three strains of *L. meyeri* by activation of the β-galactosidase system in LBg agar plate bioassays (Figure 1). A volume of at least 5 mL at a 2.4 × 10^8^ leptospires/mL concentration or higher was necessary for detection of AHL by *A. tumefaciens* NTL4.

**Figure 2:**
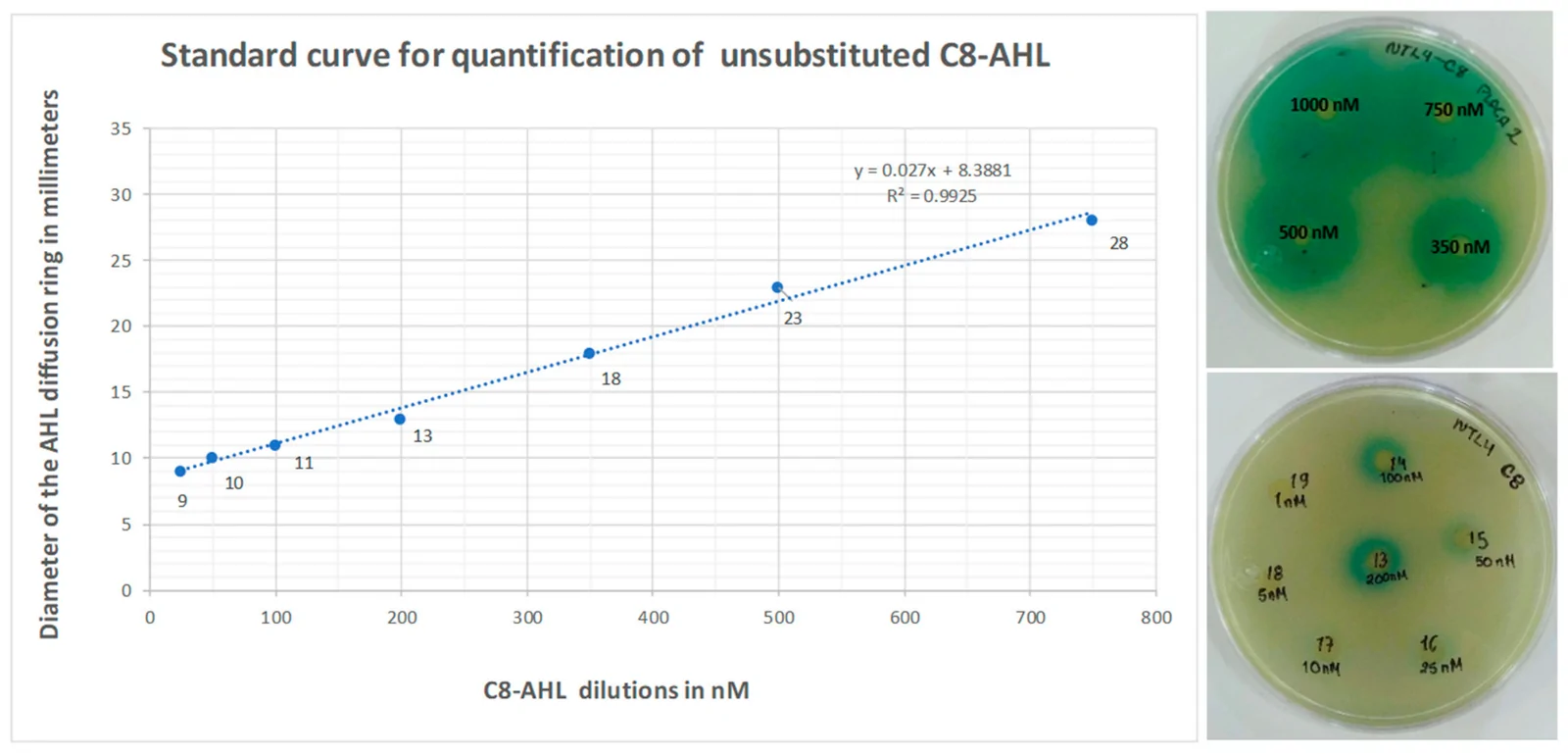
Establishment of the standard curve for quantification of unsubstituted C8-AHL. Bioassay results for dilutions of C8-AHL (nM) versus its corresponding diffusion ring diameter (mm), detected by X-gal hydrolysis. The values represent the means of three replicates and were analyzed using Pearson’s correlation coefficient. The hydrolysis of X-gal with the lower concentrations of the C8-AHL standard is better appreciated in the color images. C8-AHL concentration: 750 nM = 28 mm Ø, 500 nM = 25 mm Ø, 350 nM = 18 mm Ø, 200 nM = 13 mm Ø, 100 nM = 11 mm Ø, 50 nM = 10 mm Ø, and 25 nM = 9 mm Ø.

Under our working conditions, the minimum concentration of C8-AHL detected by the *A. tumefaciens* NTL4 biosensor strain was 25 nM of C8-AHL; the size of the AHL diffusion ring (X-gal hydrolysis) on the soft agar assay was directly proportional to the C8-AHL concentration (R^2^ = 0.9925) (Figure 2).

### 3.2. Partial Characterization of the AHL by Thin-Layer Chromatography (TLC)

**Figure 3:**
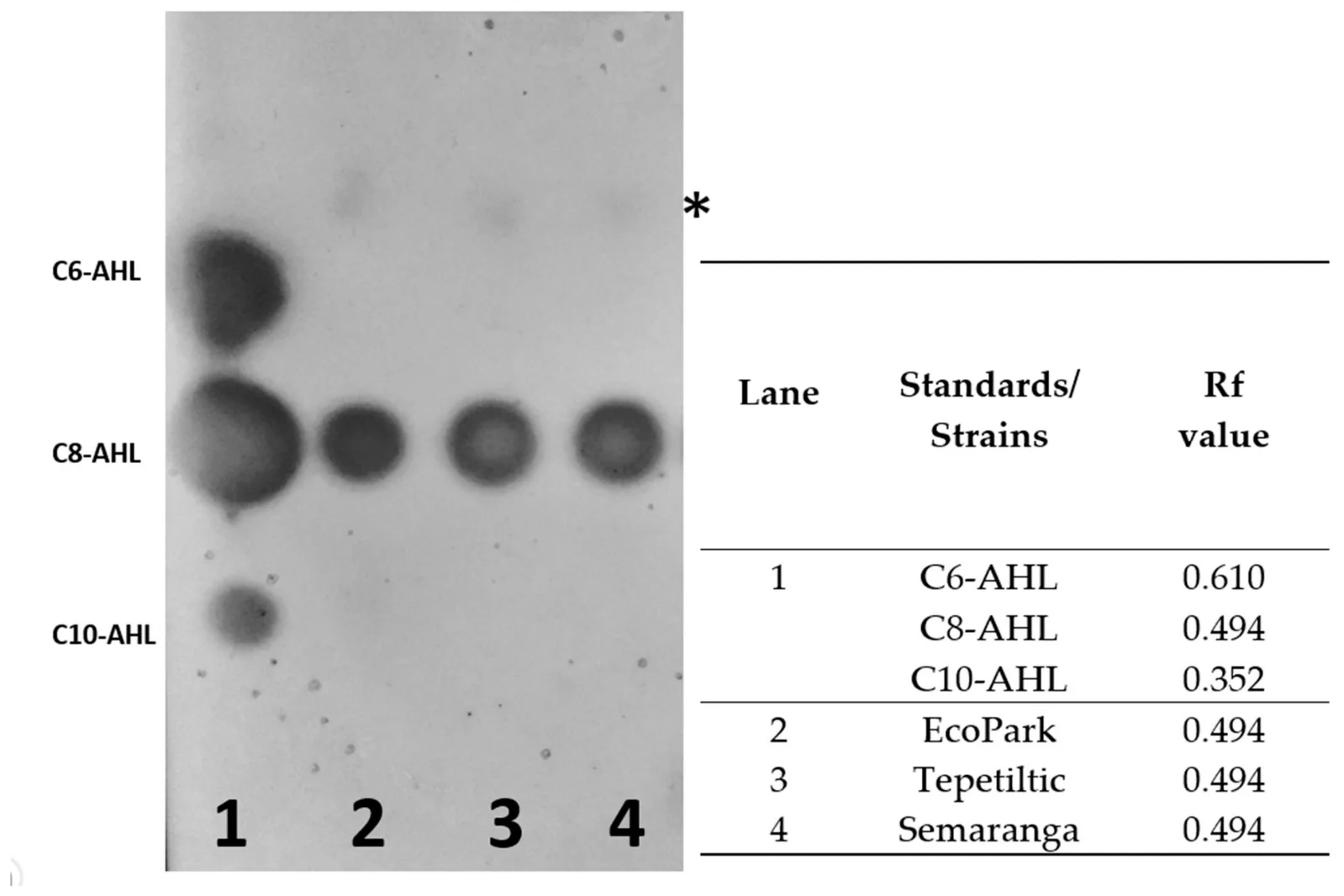
Thin-layer chromatography of unsubstituted AHL standards and the supernatant culture extracts from the *Leptospira meyeri* strains. Lane 1. Spots obtained from the synthetic standard C6-AHL 100 µM, C8-AHL 10 µM, and C10-AHL 200 µM. Lane 2. Spots obtained from strain EcoPark. Lane 3. Spots obtained from strain Tepetiltic. Lane 4. Spots obtained from strain Semaranga. The active compound spots with the highest biosensor response in each strain presented Rf values of 0.494, corresponding to the C8-AHL standard, showing that these strains are likely to produce C8-AHL. A second faint spot was also detected in the extracts of the three *Leptospira* strains with an Rf value of 0.684 that was not identified in this work (*).

Partial characterization of the AHL was done based on the mobility of the spot observed in the thin-layer chromatography assays, in comparison with the spots of the synthetic standards. *Leptospira* produced two detectable signal molecules with different chromatographic patterns, consistent between the three *Leptospira* strains. The autoinducer molecules produced by each *Leptospira meyeri* strain showing the highest concentration had a similar retention factor (0.494) to that of the synthetic autoinducer C8-AHL. The unsubstituted AHL forms obtained from the *Leptospira* culture extracts produced circular spots with sharp edges, like the synthetic standard, so we conclude the analyte exhibited chromatographic properties consistent with those of the C8-AHL standard. The compound with the lowest biosensor response was not identified in this work; the Rf of 0.684 did not correlate with the Rf values observed in the synthetic molecules used as standards in the TLC technique (Figure 3).

### 3.3. IR Results 

In the infrared spectrum of the C8-AHL standard, shown in the hydrogen-heteroatom bond vibration assay, a medium-intensity peak at 3314 cm^−1^ corresponded to the stretching vibration of the secondary amine N-H bond. In addition, three intense peaks (2953, 2923, and 2853 cm^−1^) corresponded to the stretching C-H bonds of the aliphatic groups (CH-, CH_2_-, and CH_3_-) present in this standard. In addition, two peaks corresponding to the carbonyl (C=O) lactone stretching group at 1775 and 1731 cm^−1^ were observed, and the peaks observed at 1713 and 1681 cm^−1^ corresponded to the stretching vibration of the secondary amide C=O group. Furthermore, at 1644 cm^−1,^ the peak of the bending vibration of the N-H bond was observed, and in the range of 1455 to 1362 cm^−1^, the peaks of the C-H bending vibration were observed. On the other hand, at 1172 cm^−1^, a medium-intensity peak corresponding to the stretching (CO) bond, and at 1031 cm^−1,^ the peak corresponding to the stretching (C-N) bond were observed. Finally, at 802 cm^−1,^ the peak of the bending vibration of the C-H bond was observed (Figure S1).

The same pattern of peaks was observed in the extract from the EcoPark isolate, corresponding to the C8-AHL standard. Although the presence of the peak corresponding to the N-H bond (s) was not observed, this might be the result of the presence of moisture or solvent (O-H). The bending vibrations of the N-H bond in the region of 1650–1580 cm^−1^ and the peak corresponding to the bending vibration of the C-N bond at 1250–1020 cm^−1^ were observed, confirming the presence of C8-AHL in the EcoPark isolate (Figure S2).

Concerning the spectra from the Tepetiltic isolate, the characteristic peaks of the C-H bonds (3000–2840 cm^−1^) were observed in the infrared spectrum, indicating the presence of aliphatic groups. The broad peak observed in the range of 3220–3363 cm^−1^ that corresponds to the stretching vibration of the O-H bond may be due to the solvent or to some additional compound with hydroxyl groups present in the samples. In the 1730–1715 cm^−1^ region, the peak corresponding to the lactone stretching C=O bond was observed. However, the characteristic peaks of the amide stretching C=O (1680–1650 cm^−1^) and bending N-H (1650–1580 cm^−1^) were not observed, but the peak that may correspond to the bending C-N bond was observed, indicating the presence of a trisubstituted amine (Figure S3).

In the Semaranga spectrum, no peaks were observed between 3100 and 3500 cm^−1^, and only the aliphatic stretching C-H bond peaks (3000–2840 cm^−1^) were observed. Also, the peaks of the lactone stretching C=O bond vibration were observed, which appeared in the range 1713–1730 cm^−1^, and the peaks of the C-O bond (1300–1163 cm^−1^). However, neither the C=O stretching peak in the range 1650–1680 cm^−1^ nor the N-H bending vibration peak in the range 1650–1580 cm^−1^ corresponding to the amide group were observed, confirming that C8-AHL is not present in these samples or may be present in undetectable concentrations (Figure S4).

A comparison of the IR spectra of the C8-AHL standard and culture supernatant extracts from EcoPark, Tepetiltic, and Semaranga is shown in Table 1.

### 3.4. HPLC-MS/MS

**Figure 4:**
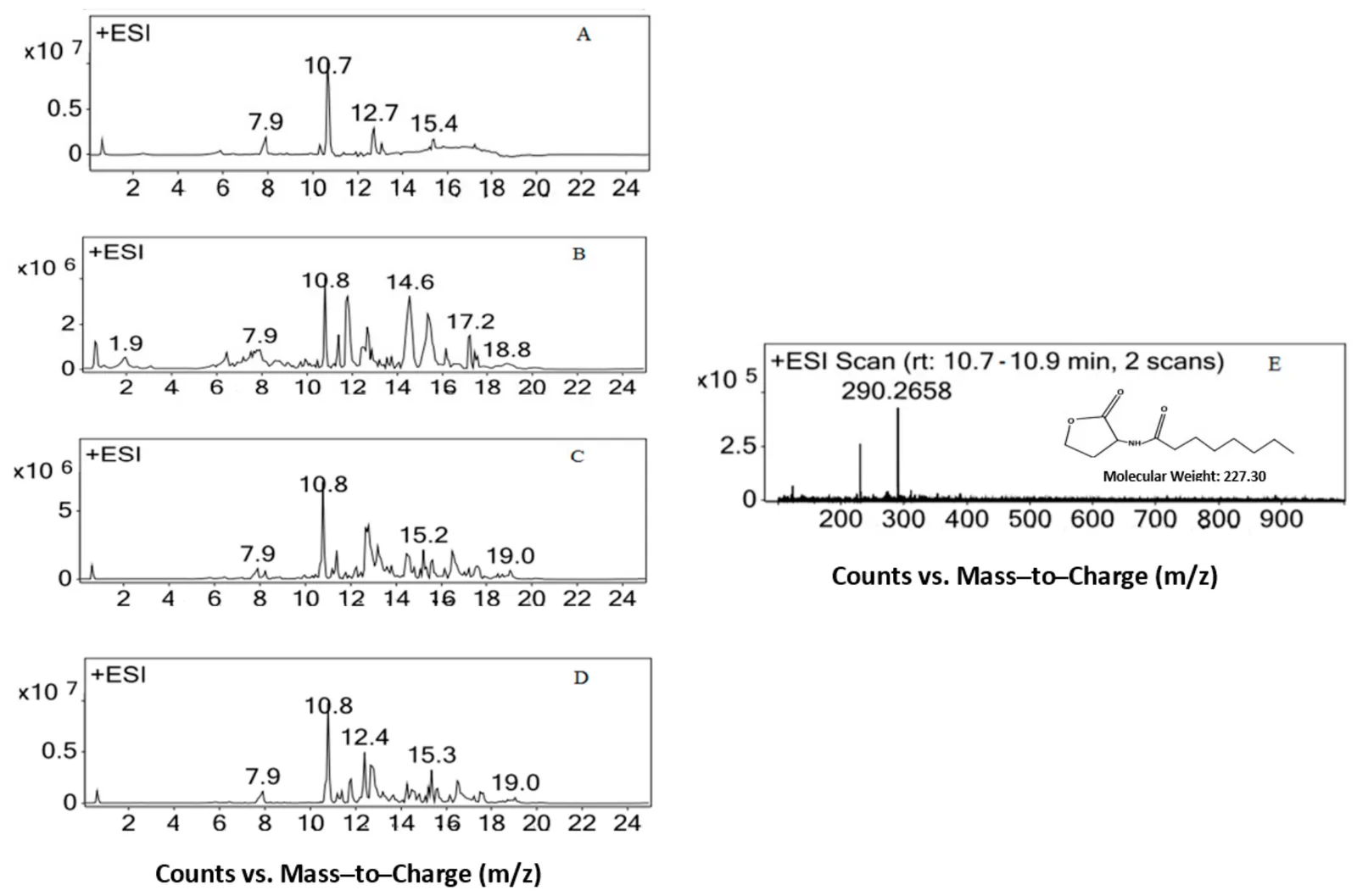
HPLC-MS/MS analysis of C8-AHL standard and active compounds obtained from extracts of a supernatant of a culture of *Leptospira meyeri* (partial purification from a preparative TLC plate). Left chromatograms: total ion chromatograms indicating the retention time of C8-AHL standard (**A**), EcoPark (**B**), Tepetiltic (**C**), and Semaranga (**D**) strains. The right spectrum shows the extracted ion for *m*/*z* 290.2658 of the C8-AHL standard (**E**); the variation between the mass-to-charge ratio (MM 227.30 to 290.26) was linked to the quasimolecular ion [M + Na + K + H]^+^; adducts are frequently generated by the employed ionization procedure.

The HPLC-MS/MS analysis for the synthetic standard C8-AHL (MM 227.30 g/mol) showed a peak with a mass-to-charge ratio (*m*/*z*) of 290.2658 (t_R_ = 10.7 min). The chromatograms of all analyzed *Leptospira* extracts revealed they were complex samples, showing an average of 30 chromatographic peaks for each sample. However, a 10.7 min ± 0.1 retention time peak was detected in all chromatograms. In addition, this conserved peak was similarly detected in the synthetic standard C8-AHL when analyzed by mass spectrometry, showing the same quasimolecular ion [M + Na + K + H]^+^ = 290.2658 *m*/*z* (Figure 4). For each *Leptospira* extract in which the quasimolecular ion was detected, the mass defect was confirmed to be no greater than 10 ppm, and the MS/MS spectrum corresponded to that obtained for the standard (Figure S5).

## 4. Discussion

A large number of AHL-dependent QS have been identified in various members of the Proteobacteria group using methods based on a bacterial biosensor system. However, beyond Proteobacteria, the production of AHLs has also been described in other phyla. For example, in Cyanobacteria, the genus *Gloeothece* has been observed to produce N-octanoyl-homoserine lactone (C8-AHL) [23]; *Tenacibaculum maritimum,* a member of the group Bacteroidetes, produces N-butyryl-homoserine lactone (C4-AHL) [24], and the Archaea, *Methanosaeta harundinacea* synthesized a new class of carboxylated AHL with 10 to 14 carbon atoms in the acyl chain [25,26]. The production of C3-oxo-octanoyl-homoserine lactone (3-Oxo-C8-AHL) has even been demonstrated in a novel strain of *Exiguobacterium* sp., a marine Gram-positive bacterium [27].

In the phylum Spirochetes, AHL-dependent QS has not been described yet; however, the first description of interspecies AI-2-dependent QS was done for *Borrelia burgdorferi*, the causal agent of Lyme disease or borreliosis [28]. To function properly, many proteins, nucleic acids, and other molecules need to be modified by the chemical addition of a methyl group, and S-Adenosyl methionine (SAM) is the principal methyl group donor (by the activated methyl cycle). These cellular methylation reactions produce S-adenosylhomocysteine (SAH), a toxic byproduct, which acts as a competitive inhibitor of the same methylation reaction. The detoxification of SAH is made through two pathways: a one-step mechanism utilizing SAH hydrolase, resulting in the production of adenosine and homocysteine; and a two-step mechanism by Pfs/LuxS (Pfs nucleosidase/S-Ribosylhomocysteine liase) with subsequent production of homocysteine and the autoinducer 2 (AI-2) [29].

*Borrelia burgdorferi* uses the Pfs/LuxS pathway, a two-step process, for the detoxification of SAH, a route essential for AI-2 production [30]. For the genus *Leptospira*, it has been mentioned that its genome contains a *metK* homolog but lacks the *luxS* and *pfs* genes, so that it cannot produce the intermediate molecules necessary for the formation of AI-2 [30,31]. On the other hand, *Treponema pallidum* and *Treponema denticola,* the causative agents of syphilis and periodontal disease, respectively, have been reported to possess in their genomes genes coding for the MetK and Pfs enzymes but lack homologous genes coding for the LuxS enzyme [32]. A subsequent phylogenetic study mentioned that the *luxS* gene sequence of *Borrelia burgdorferi* is a sister group of the *luxS* gene of *Clostridium acetobutylicum*, suggesting that bacteria can acquire regulatory genes through horizontal gene transfer, which seems to explain why the AI-2-dependent QS is limited only to the genus *Borrelia* [17,31]. In this regard, in silico analysis in GenBank confirmed that no homologous *luxS* genes were found in the *Leptospira* genomes [31,33].

Currently, three families of non-homologous enzymes capable of synthesizing AHL-type autoinducers (LuxI, LuxM/AinS, and HdtS) have been identified. Phylogenetic analyses indicate that *Leptospira* has no genes coding for synthetases of the LuxI family. The LuxI synthetase family is the best-studied, and its homologs have been described in many Gram-negative bacteria. In contrast, the two homologous LuxM/AinS proteins have been described only in *Vibrio* and related species [34]. A third family was described in *Pseudomonas fluorescens*, the HdtS family that shares similarities with enzymes belonging to the lysophosphatidic acid transferase (LPAAT) family, and whose main metabolic roles are in glycerolipid biosynthesis. LPAATs are critical in both membrane lipid biosynthesis and storage lipid metabolism [35,36,37].

Homologs of HdtS are ubiquitous among bacteria and eukaryotes; however, the enzymatic mechanism of HdtS-catalyzed AHL synthesis remains uncharacterized. Furthermore, the features of the HdtS enzymes that are required for AHL production are unknown, but there are homologs in organisms that do not produce AHLs. Therefore, this bacterial enzyme family appears to have two functions: acylation of lysophosphatidic acid and AHL synthesis [38]. It is worth mentioning that several studies have reported the detection of AHL-type molecules by biosensor strains in microorganisms with no genetic evidence for the presence of the LuxI or LuxM AHL synthetases, suggesting that AHL synthesis may occur, possibly mediated by an HdtS or other alternative pathways [39].

In different species of *Leptospira*, we found homologs of HdtS, a putative N-acylhomoserine lactone synthase protein. HdtS from *L. meyeri* (WP_238761268) has 27.17% identity with HdtS from *Pseudomonas fluorescens* (AF286536_3); despite the low similarity, the NHQS and PEGTR motifs characteristic of the 1-acylglycerol-3-phosphate O-acyltransferase enzyme family (2.3.1.51) were found. Even with the above, we believe it is very daring to hypothesize that C8-AHL is synthesized by HdtS, but it is not conclusive, as *Leptospira* have several proteins with acyltransferase motifs. Complementary in silico analyses should be conducted not only on the AHL synthase but also on other genes related to the QS in future investigations.

In this work, using the biosensor *Agrobacterium tumefaciens* strain NTL4, together with TLC, IR, and HPLC-MS/MS, we provide evidence of N-Octanoyl-homoserine lactone (C8-AHL) production in cultures of *Leptospira meyeri*.

The *Agrobacterium tumefaciens* strain NTL4 is a sensitive and broad-spectrum biosensor that has the *lacZ:: traG* fusion coding for the β-galactosidase enzyme, which in turn is regulated by the QS and can detect AHLs with medium to long acyl chains (6 to 14 carbons), with or without hydroxy or oxo substitutions [40]. When the AHLs are added exogenously, they activate the *lacZ::traG* fusion, which in turn is detectable by the appearance of a blue halo produced by the hydrolysis of the X-Gal substrate [41].

In this work, by using the *A. tumefaciens* strain NTL4 as a biosensor, we detected autoinducer molecules capable of activating the β-galactosidase system in culture extracts of three strains of *Leptospira meyeri*; the reference strain Veldratem 173 serovar Semaranga, and two field isolates: Tepetiltic and EcoPark. The joint use of the biosensor strain and the TLC technique provided us with a rapid and direct visual marker for partial identification of autoinducer molecules produced by *Leptospira meyeri*. The detected molecule with the highest concentration showed a retention factor similar to that of the synthetic autoinducer C8-AHL (0.494). In addition, TLC plates also showed another active molecule with a higher retention factor that was not identified in this work; the Rf of 0.684 did not correlate with the Rf values observed in the synthetic molecules used as standards in the TLC technique; that TLC profile was consistent across the three *Leptospira meyeri* strains analyzed. These findings suggest that the genus *Leptospira* could produce more than one type of AHL. It has been reported that numerous genera of Gram-negative bacteria are capable of producing more than one type of AHL [42,43,44].

Regarding the identification of an autoinducer molecule, our findings are in agreement with previous works, where the use of biosensor strains and the TLC technique producing circular spots with well-defined and sharp edges similar to the synthetic standard is interpreted as the presence of an autoinducer molecule with a similar structure to that of the synthetic standard [19]. So we assume that the detected molecule in the analyzed supernatants corresponded to an unsubstituted C8-AHL or a compound with a similar structure. The TLC technique does not allow unequivocal identification of different types of AHLs [19,40], but its chromatographic properties helped us to tentatively identify the type of AHL produced by *Leptospira meyeri*. Therefore, the Rf value is used to determine the length of the side chain, as they migrate at different rates due to the differences in polarity. The shape of the spots is related to the substitution present at carbon 3 of the side chain; thus, 3-oxo-substituted AHLs produce spots with fuzzy-edged tails, and 3-hydroxy-substituted AHLs migrate with the same mobility as their 3-oxo analogs, but the spots do not tail [41].

Despite the advantages offered by using bacterial biosensors, they also have limitations, so caution should be exercised when interpreting the data obtained with them. It has been mentioned that supernatant extracts might contain compounds with a similar structure to AHL that could interfere [41] with or activate the response of biosensors, such as decetopiperazines (DKPs) [45]. Additionally, the absence of positive responses may be attributed to a low concentration of the autoinducer that is undetectable by the biosensor.

The presence of AHL-type autoinducers in the extracts of the *L. meyeri* strains was confirmed by infrared spectroscopy. The infrared spectrum showed the same pattern of peaks in the synthetic C8-AHL standard and in the extract of the EcoPark strain of *Leptospira meyeri*. These results support the biological activity of the autoinducer molecules of the EcoPark strain detected in the bioassays. However, in the spectrum of the Tepetiltic strain, metabolites with functional groups similar to those observed in the C8-AHL compound were detected. In contrast, the spectrum of the Semaranga strain did not detect these functional groups, which could be attributed to the low concentration of AHL in the extract (Table 1).

The HPLC-MS/MS chromatogram for the synthetic standard C8-AHL showed the most abundant peak with a mass-to-charge ratio (*m*/*z*) of 290.2658 and a retention time (tR) of 10.7 ± 0.1 min. Such a *m*/*z* 290.2658, MS/MS, and tR = 10.7 ± 0.1 min peak was also observed in the chromatograms of the three *Leptospira meyeri* EcoPark, Tepetiltic, and Semaranga strains, confirming the presence of C8-AHL in the extracts from those strains. In this study, the formation of adducts in the synthetic autoinducer C8-AHL molecule interfered with the efforts to obtain the characteristic mass spectrum of *m*/*z* 227.30. This was associated with the quasimolecular ion [M + Na + K + H]^+^, an adduct commonly formed during electrospray ionization [46].

The HPLC-MS/MS technique is more sensitive for identifying and quantifying molecules compared to the FTRI technique, which is consistent with what was observed in this work; despite its widespread application, FTIR is susceptible to spectral interference, particularly from polar solvents such as methanol, and is inherently limited in both spectral resolution and analytical sensitivity. Overlapping absorption bands can hinder the discrimination and quantification of low-abundance analytes, while FTIR is typically restricted to the analysis of major components present at concentrations above approximately 0.1% (>1000 ppm). By comparison, HPLC offers superior sensitivity and selectivity, allowing reliable detection and quantification of analytes at parts-per-billion (ppb) concentrations [47].

The concentration of C8-AHL produced by *Leptospira meyeri* can be qualitatively estimated based on standard curves obtained from the agar-diffusion bioassays. However, the TLC analysis of the same extracts showed two detectable signal molecules with different chromatographic patterns. Therefore, we concluded that the autoinducer molecule responsible for the highest biosensor response (X-gal hydrolysis zone) was the same, showing an Rf = 0.494 in the TLC analysis. However, the presence of a compound with a faint spot in the TLC analysis (Rf = 0.684) could lead to overestimating these results. Despite the lack of quantitative data, the detection of these molecules similar to the C8-AHL indicates the accumulation of a biologically active autoinducer significant for cell communication.

In a wide variety of bacteria, the *Quorum Sensing* system has been shown to regulate biofilm formation, development, and disintegration. Biofilms act as virulence factors that contribute to persistent infections and as growth and development factors that allow bacteria and other organisms to survive in hostile environments. Biofilm formation has been determined as an outstanding survival capability for bacteria, as it has been observed that more than 99% of bacterial cells exist as biofilms [48].

Microbial communities in biofilms have a survival advantage, as these bacterial assemblages are highly complex, dynamic systems where bacteria coexist, collaborate, compete, and exchange information in a coordinated manner, and directly or indirectly influence the microbial community to adapt to the changing environment [49,50].

*Leptospira* is a complex bacterial genus; saprophytic species successfully occupy a wide variety of soil and water habitats, while pathogenic species have developed virulence factors that allow them to infect, cause disease, and persist in host tissues (mainly, the renal convoluted tubules). The first mention of cell aggregates or possible biofilm formation was made in 2003 [51]; subsequently, a study in 2008 described biofilm produced by *Leptospira* [52].

It has also been proposed that, in *Leptospira,* the *Quorum Sensing* system plays an important role in the regulation of biofilm formation in both the environment (survival and dissemination) and in animal host tissues (chronicity of the disease) [7]. A more recent study demonstrated that *Leptospira* follows the c-di-GMP pathway to produce resilient biofilms and plays a key role in motility [53]. Subsequent work described the formation of biofilms by pathogenic *Leptospira* in the renal tubules of naturally infected *Rattus norvegicus* (Norway rats). A positive correlation was observed between the intensity of the tubule colonization and biofilm formation, where the higher the number of colonized tubules, the higher the probability of renal biofilm formation by pathogenic *Leptospira*. On the other hand, considering the number of colonized renal tubules as a measure of *Leptospira* population within the organ could indicate that in vivo *Leptospira* biofilm formation depends on the number of leptospires during renal colonization, which in turn suggests the presence of a *Quorum Sensing* mechanism [54]. However, the mechanisms of biofilm binding and maturation are still unknown, so it has been suggested that studies such as transcriptomic analysis of early and late biofilms and *Quorum Sensing* would help fully understand the mechanisms of biofilm regulation and formation in *Leptospira* [55].

In this work, we report the presence and identification of the N-Octanoyl-DL-homoserine lactone (C8-AHL) autoinducer in *Leptospira meyeri* culture supernatants. This finding is the first observational evidence of the presence of a QS in *Leptospira*. Future research should be directed toward describing this system, as well as its implication in the regulation of biological functions in *Leptospira*.

## Figures and Tables

**Table 1:** Comparison of the IR spectra of the C8-AHL standard and culture supernatant extracts from the isolates EcoPark, Tepetiltic, and Semaranga strain of *Leptospira meyeri*. S = stretching, f = flexion, oop = out of plane, b = broad, oor = out of range.

Bond (cm^−1^)	C8-AHL (STD)	EcoPark	Tepetiltic	Semaranga
N-H (s) (3350–3310)	3314	-	-	-
O-H (s) Solvent or Water	-	3363 (b)	3220 (b)	-
C-H (s) (3000–2840)	2953 2953 2853	2927 2871 2857	2955 2921 2852	2955 2922 2852
C=O (s) lactone (1730–1715)	1775 1731 1713	1713	1741 1709	1711
C=O (s) amide (1680–1650)	1681	1665	-	-
N-H (f) (1650–1580)	1644	1560 (b) (oor)	-	-
C-H (f) (1465–1375)	1455 1377	1455 1378	1459 1376	1459 1376
C-O (1300–1163)	1260 1172	1249 1220	1259 1162 1097	1260 1162 1112
C-N (f) (1250–1020)	1031	1044	1025	-
C-H (oop) (900–790)	802	8553	802	803

## Data Availability

All data are available upon request from Alejandro de la Peña-Moctezuma, delapema@unam.mx, and Carlos Alfredo Carmona-Gasca, carmonagasca@uan.edu.mx.

## Supplementary Materials

The following supporting information can be downloaded at: https://www.mdpi.com/article/10.3390/microorganisms14081806/s1, Figure S1: FT-IR spectra of C8-AHL standard; Figure S2: FT-IR spectra of the extracts from the *Leptospira meyeri* EcoPark isolate; Figure S3: FT-IR spectra of extracts from the *Leptospira meyeri* Tepetiltic isolate; Figure S4: FT-IR spectra of extracts from the *Leptospira meyeri* Semaranga strain; Figure S5: MS/MS spectra of C8-AHL standard.
