# Supplementary material for: Identification of Quorum Sensing Molecules of N-Acyl-Homoserine Lactone in Leptospira Strains Supernatants
Source: Microorganisms. 2026 Aug 16;14(8):1806. doi: 10.3390/microorganisms14081806 (PMC13515436; doi:10.3390/microorganisms14081806)
Supplement: Supplementary file 1 [file microorganisms-14-01806-s001.zip › microorganisms-4473907-supplementary.pdf]

Supplementary material

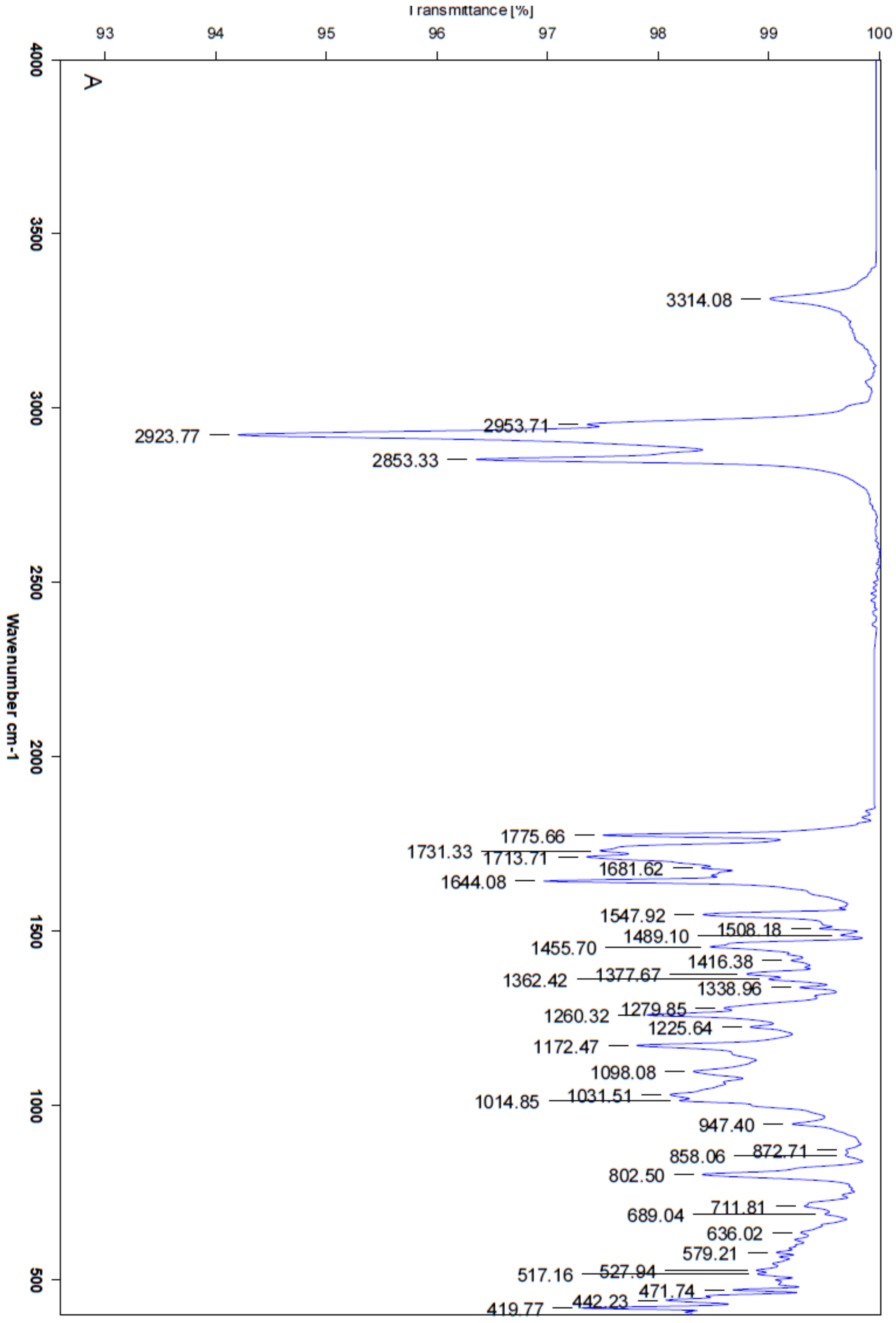

Figure S1: FT-IR spectra of C8-AHL standard

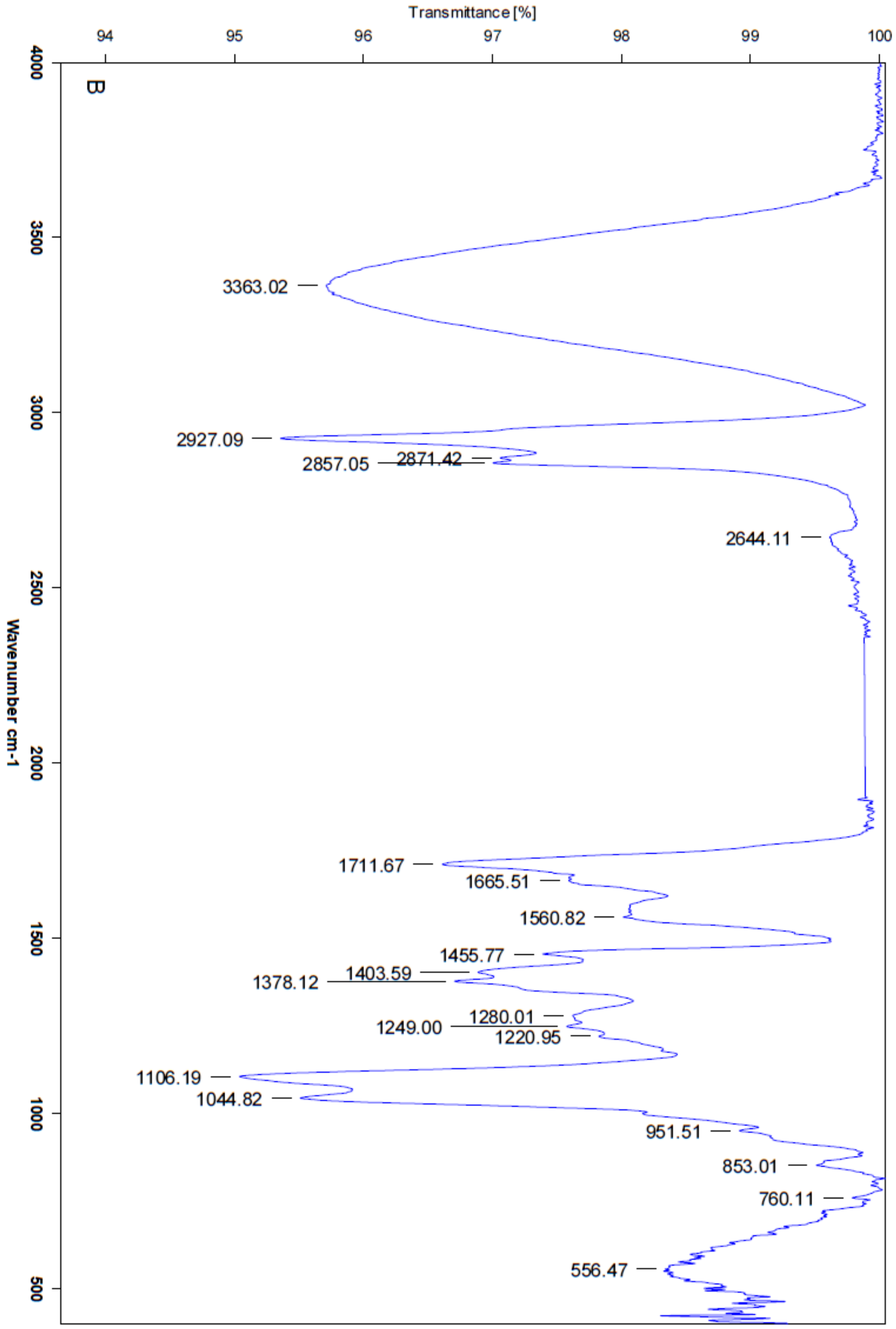

Figure S2: FT-IR spectra of the extracts from the *Leptospira meyeri* EcoPark isolate.

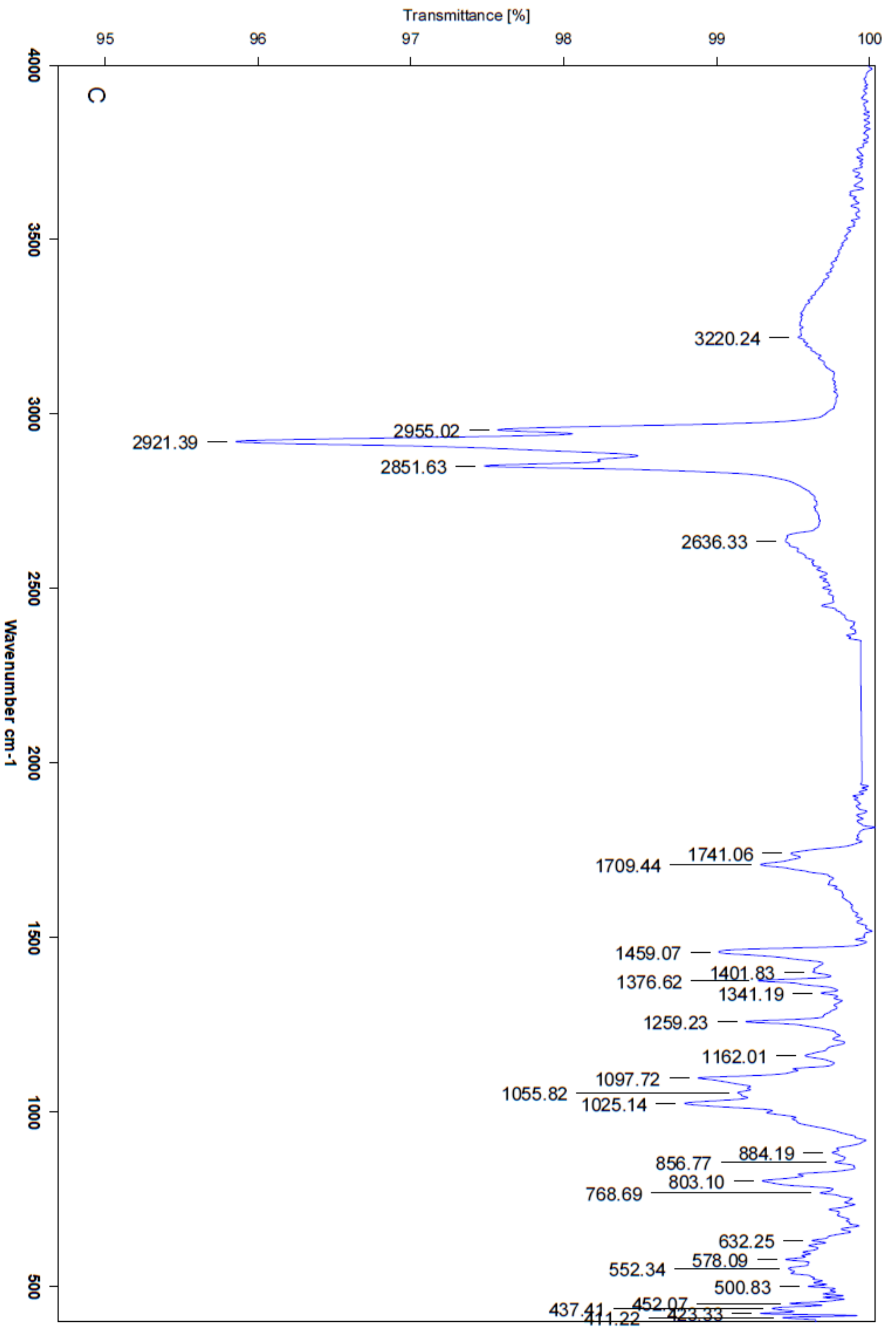

Figure S3: FT-IR spectra of extracts from the *Leptospira meyeri* Tepetiltic isolate.

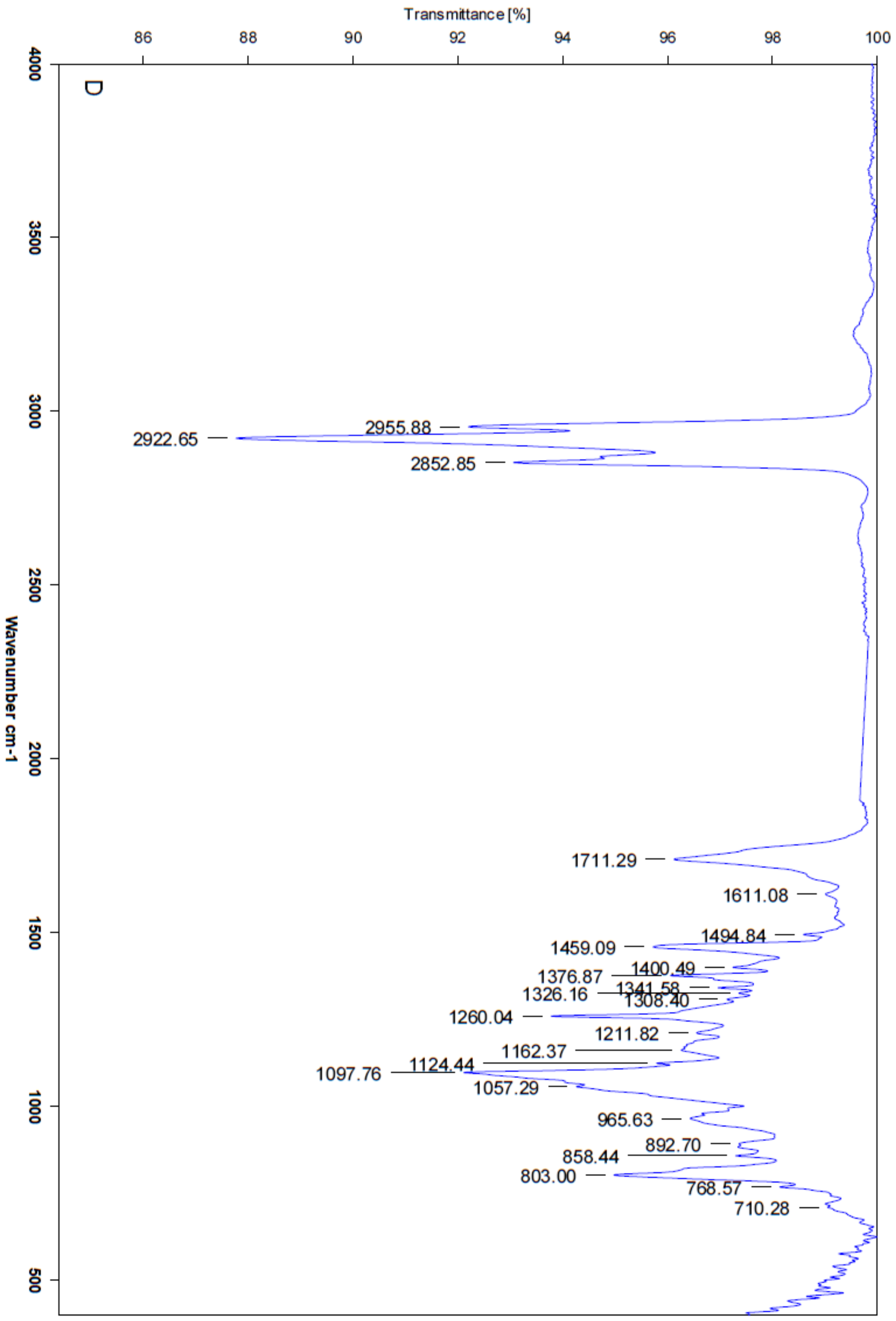

Figure S4: FT-IR spectra of extracts from the *Leptospira meyeri* Semaranga strain

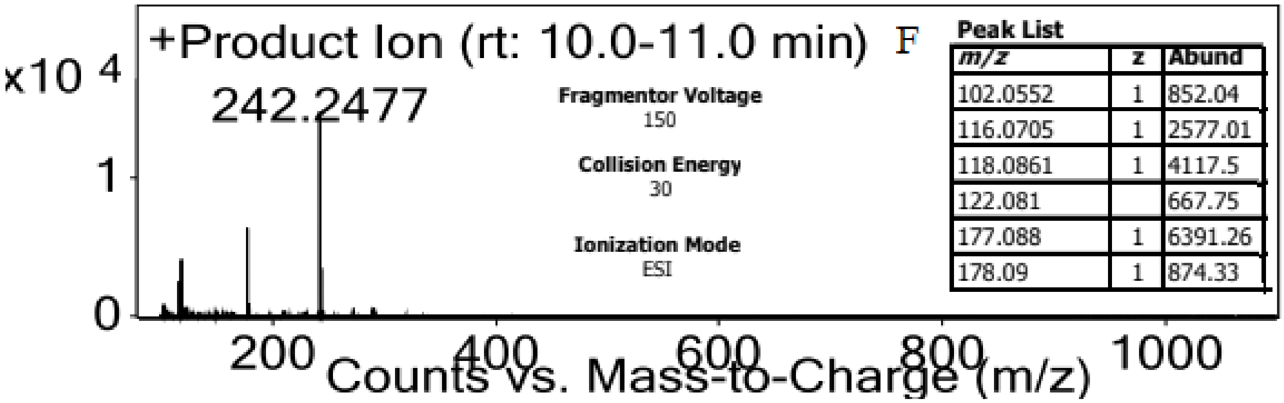

Figure S5: MS/MS spectra of C8-AHL standard
